# Selected serum cytokines and nitric oxide as potential multi-marker biosignature panels for Parkinson disease of varying durations: a case-control study

**DOI:** 10.1186/s12883-019-1286-6

**Published:** 2019-04-06

**Authors:** Dilini Rathnayake, Thashi Chang, Preethi Udagama

**Affiliations:** 1 Department of Zoology and Environment Sciences, Faculty of Science, University of Colombo, Colombo 3, Sri Lanka; 2 Department of Clinical Medicine, Faculty of Medicine, University of Colombo, Colombo 8, Sri Lanka

**Keywords:** Parkinson disease, Cytokines, Nitric oxide, Serum biomarkers, Sri Lanka

## Abstract

**Background:**

Dopaminergic neuronal loss begins years before motor symptoms appear in Parkinson disease (PD). Thus, reliable biomarkers for early diagnosis and prognosis of PD are an essential pre-requisite to develop disease modifying therapies. Inflammation-derived oxidative stress is postulated to contribute to nigrostriatal degeneration. We evaluated the role of selected serum immune mediators (IFNγ, TNFα, IL-10, and NOx) in PD progression and estimated their usefulness in preclinical diagnosis.

**Methods:**

This case-control study recruited 72 PD patients with varying disease durations (< 1-year, *n* = 12 patients; 1–3 years, *n* = 30; > 3 years, n = 30) and 56 age- and gender-matched controls (26 with other neurological disorders as disease controls, and 30 healthy controls). Serum cytokine levels and NOx quantified using Sandwich Enzyme Linked Immunosorbent Assay kits, and the Griess test, respectively, were evaluated for diagnostic accuracy of optimal marker combinations by the CombiROC method. PD patients were clinically evaluated for motor and non-motor symptoms, and staged based on Hoehn and Yahr (H-Y) scale.

**Results:**

A significant increase in serum IFNγ and IL-10 was observed in PD compared to healthy controls (*p* < 0.001). The Th1: Th2 (IFNγ: IL-10) cytokine ratio was higher in PD of 3–12 years compared with PD < 1 year (p < 0.001). Highest levels of NOx manifested during early PD (1–3 years) through a subsequent decline with disease duration. TNFα level was highest at PD onset. A low serum NOx level was associated with cognitive impairment (*p* = 0.002). The potential of using multi-biomarker panel, IFNγ, IL-10 and TNFα, for detection of PD onset was evident (sensitivity [SE] = 83.3%, specificity [SP] =80.4%, area under curve [AUC] = 0.868), while for early and late PD the multi-biomarker signature of IFNγ, IL-10 and NOx appeared to be more promising (SE = 93.3%, SP = 87.5%, AUC = 0.924).

**Conclusion:**

A Th1 cytokine-biased immune response predominates with PD progression. Both IFNγ and IL-10 are involved in disease severity. However, TNFα-mediated neurotoxicity appears to occur in early PD.

**Electronic supplementary material:**

The online version of this article (10.1186/s12883-019-1286-6) contains supplementary material, which is available to authorized users.

## Background

A broad spectrum of neurodegenerative diseases are associated with chronic inflammation in the central nervous system (CNS) including Parkinson disease (PD) [1, 2]. This inflammation-derived oxidative stress is postulated to be a contributor in degeneration of the nigrostriatal pathway hastening PD progression [3]. PD accompanied by neuroinflammation is mediated by activated brain-residing astrocytes, microglial cells and infiltrating T lymphocytes. Since gliosis and astrocytosis release neurotrophic factors such as inflammatory cytokines and reactive oxygen and nitrogen species (ROS and RNS) in vivo, these effects could be transferred to the peripheral immune system due to the changes in the micro-environment during its pathological cascade [4, 5].

Given that PD is a neurodegenerative disease, it is imperative that disease modifying strategies are administered at an early stage of the disease if it is to have any impact on the disease course [6]. In this context, it is crucial to identify clinically useful reliable biomarkers that will be able to identify PD long before the manifestations of motor symptoms, which is a neuropathologically advanced stage at which PD is diagnosed currently.

Abnormalities in peripheral immune functions in patients with PD include changes in lymphocytic subpopulations in blood, deviation of T-lymphocyte subsets, impaired production of IL-2 and higher production of IFNγ by peripheral blood mononuclear cells (PBMC) that include elevation of some cytokines such as IL-2, IL1-β, IL-10, IL-4, IL-6 and TNF-α in serum or plasma [7–11].

Due to current limitations in neuroimaging methods to measure pathologic changes in dopaminergic brain structures, the focus on the use of serum biomarkers for diagnosis and prognosis of PD appears to be the thrust of current research [12]. These biomarkers can be categorised based on trait, state and rate. A trait biomarker indicates disease susceptibility, while a state biomarker is diagnostic of disease, and a rate biomarker tracks disease progression [13]. To date, clinical diagnostic tests for PD based on biochemical analysis of blood are unavailable. There is, however, a recent upsurge in studies focusing on the development of blood-based biochemical markers diagnostic of PD prior to clinical diagnosis, as the latter is based merely on motor symptoms [14, 15]. The need and quest for serum immunological mediators as predictive markers of risk of PD, and also as diagnostic and prognostic biomarkers of the disease, is imperative [11, 16, 17].

In this study, we assayed a panel of serum immune-mediators, i.e. cytokines IFNγ, TNFα and IL-10, and derivatives of nitric oxide (NOx) from a PD cohort of varying disease durations, with age and gender matched controls. The relationship of serum levels of these mediators with disease progression and motor and non-motor complications of PD were established. We aimed to assess the potential of these mediators as state and/ or rate biomarkers for PD; the accuracy of these immune-mediators as multi-biomarker panels was evaluated using the CombiROC method [18].

## Methods

### Subjects

During a period of six months (June to December 2016), we recruited patients with idiopathic PD of varying chronological history from the day of their first motor symptom and/or clinical diagnosis: (i) < 1-year PD, (ii) 1–3 years PD and (iii) 3–12 years PD, from selected Neurology clinics in the district of Colombo, Sri Lanka. Age- and gender-matched healthy adult volunteers with the same ethnic origin and area of residence served as ‘normal, healthy controls’ while, individuals with other neurological disorders other than PD (i.e. stroke, myasthenia gravis, multiple sclerosis, Guillain-Barre syndrome, etc.), were enlisted as ‘disease controls’. All patients were examined by a single neurologist (TC). PD was diagnosed based on established clinical criteria [19]. Patients with evidence of atypical parkinsonian syndromes, secondary parkinsonism or with current infections, associated chronic inflammatory diseases including connective tissue diseases and multisystem inflammatory disorders, malignancies or patients on immunosuppressive drugs were excluded from the study.

All PD patients were clinically evaluated for motor and non-motor symptoms and staged based on Hoehn and Yahr (H-Y) scale for motor impairment and disability [20] (0 represents asymptomatic PD whereas 5 denotes severe PD). A score of < 26/30 on ‘Montreal Cognitive Assessment’ (MoCA) was considered to represent cognitive impairment. Socio-demographic data were collected using an interviewer-administered questionnaire. All patients recruited were randomly chosen, based on their order of attending the neurology clinic without any bias and were later divided into the three groups as stated above, during data analyses.

### Blood collection and serum separation

Five ml of venous blood from each participant was collected, allowed to clot for 15 min at room temperature prior to centrifugation at 2000 rpm for 15 min [21]. Serum was separated and aliquots were stored at -20 °C until analysis.

### Detection of serum levels of cytokines and NOx

#### Serum cytokines

Serum cytokine levels of IFNγ, TNFα and IL-10 were assayed using human sandwich Enzyme Linked Immunosorbent Assay (ELISA) kits as per manufacturer specifications (Becton & Dickinson OptEIA™, USA). These cytokines were detected as antigens in serum using capture and detection monoclonal antibodies against specific cytokines.

Briefly, a standard 96-well microtitre plate (Immulon™ 2 HB, High Binding, USA) was used for assaying the cytokine levels in serum. The microtitre wells of the plate coated with capture antibody was incubated at 4 °C overnight. Upon washing, the non-specific binding was blocked by adding assay diluent (PBS in 10% FBS). The plate was incubated at room temperature (RT) for 1 h. The standards and the serum samples (diluted in assay diluent) were dispensed into wells and after 2 h of incubation, the detection antibody with the biotin-streptavidin enzyme conjugate was added into each well. After 1 h, on adding the substrate solution, the plate was sealed and incubated at RT in the dark. The stop solution (2N H_2_SO_4_) was added, and the optical density (OD) of each well was read at 450 nm using a microplate reader (BIO-RAD Model 680; Microplate Reader, USA). The concentration of IL-10, IFNγ and TNFα in each serum sample was calculated using standard curves of known cytokine concentrations.

#### Nitric oxide mediators (NOx)

The NOx levels in serum samples were assayed by the Griess reaction assay [22]. Briefly, the serum samples were deproteinised using 20 μl of zinc sulphate (15 mg/ml) and centrifuged at 10,000 rpm for 10 min. Vanadium (III) Chloride was used to convert NO_3_^−^ in serum to NO_2_^−^. With the addition of the Griess reagent, the resultant reddish-pink azo dye was quantified colorimetrically. The serum NOx levels were determined from a linear standard curve established by 0–100 μmol/dm^3^ of sodium nitrite.

### Questionnaire

The motor and non-motor symptoms of PD patients were assessed and recorded with their past medical history using a standard questionnaire (Additional file 1). The Montreal Cognitive Assessment (MoCA) test was used to assess the cognition of PD individuals. A score of < 26/30 on MoCA was considered to represent cognitive impairment.

### Data analyses

Statistical analyses were performed using SPSS version 20.0 (IBM Corporation, New York, USA) and Graph-Pad Prism (GraphPad Software, Inc., San Diego, California, USA) after data collection and laboratory analyses of serum immune markers. The data were tested for normality distribution prior to statistical analyses. The continuous variables, not normally distributed were expressed as mean ± SD and compared using non-parametric tests. The clinical/demographic variables of the cases and controls were compared as follows; independent-samples T test for continuous variables and chi-square test for categorical variables. The mean serum immune marker levels were compared using Mann-Whitney U/Kruskal-Wallis H tests, as appropriate. The relationship between the analytes and the H-Y scale of PD were analysed using the Spearman correlation coefficient.

A receiver operating characteristic (ROC) curve evaluates accuracy of a diagnostic test i.e. to discriminate between a target disease condition from health. A combinatorial analysis of multiple biomarker signatures was carried out to determine the diagnostic accuracy of optimal marker combinations of the tested serum cytokines and NOx using the CombiROC method. This allows to determine optimal combinations of biomarkers through a combined analysis of ROC curves, considering the sensitivity (SE) and specificity (SP) of all possible markers which is implemented as a freely available web application (http://CombiROC.eu). Briefly, once the data is uploaded, the software carries out a combinatorial analysis between all analytes of cases and controls in a pair-wise manner to compute all possible marker combinations. In the second phase, it computes and evaluates the true positives to rule-out the disease status (or SE) and the true negatives to rule-in the disease status with high confidence (or SP) of all possible marker combinations analysed and select the best performing group of combos/combinations with the highest SE and SP [18].

## Results

### Participant characteristics

A total of 72 patients with idiopathic PD of varying disease durations, 30 normal healthy controls and 26 with other neurological disorders were recruited for the study. All patients were recruited from two different neurologic clinics in the Colombo district, Western Province, Sri Lanka. Most patients were on anti-parkinsonian drug therapy unless diagnosed first time by the neurophysician (*n* < 5). All participants belonged to the same ethnic group based on the same ancestry and geographic origin and socio-cultural background [23]. (Additionally, three generations of spoken language were employed as a tool of differentiation). The participants’ demographic and clinical characteristics which were significantly not different among the test groups (*P* > 0.05) are summarised in Table 1.

**Table 1:** Demographic and clinical characteristics of test and control subjects

Test groups	Parkinson Disease (PD) (*n* = 72)			Controls (*n* = 56)	
Variables	< 1 year PD (*n* = 12)	1–3 years PD (*n* = 30)	3–12 years PD (*n* = 30)	Other neurological disorders (*n* = 26)	Healthy individuals (*n* = 30)
Age (years)	64.4 (±9.9)	66.5 (±10.0)	63.9 (±9.6)	57.2 (± 8.6)	51.0 (± 9.6)
Sex (% male)	66.7%	56.7%	60.0%	30.8%	56.7%
H-Y scale	1.36 (±0.45)	2.24 (±0.96)	2.38 (±1.36)	–	–

All data are presented as mean ± standard deviation where applicable. The following statistical tests were used; independent-samples T test for continuous variables and chi-square test for categorical variables (*P* > 0.05 for all characteristics analysed)

### Levels of selected serum cytokines and NOx of PD patients

**Fig. 1:**
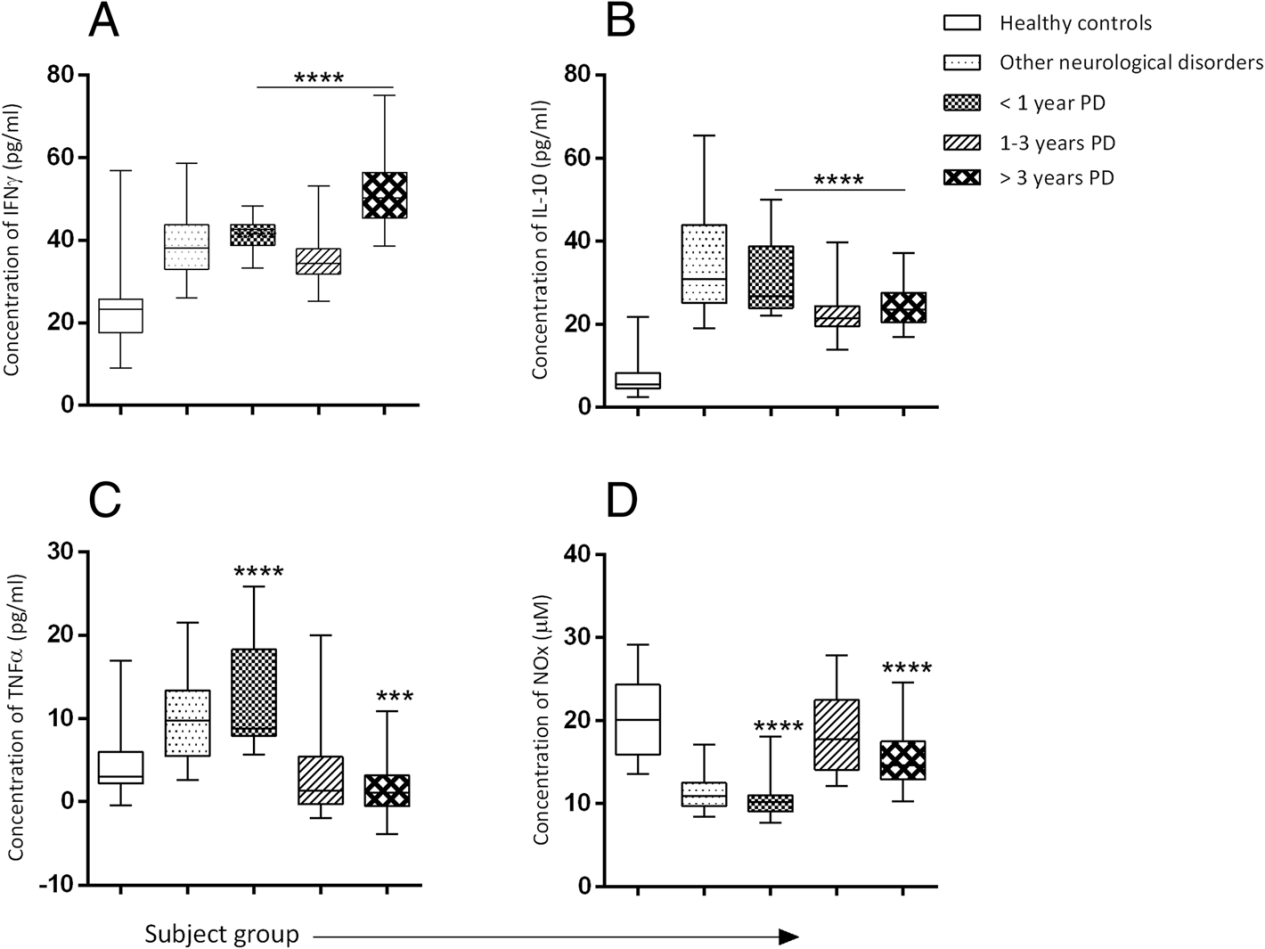
Concentrations of serum immune mediators in Parkinson patients with varying disease durations compared with controls (**a**) Mean serum IFNγ concentration in pg/ml (< 1-year PD = 41.76[±3.92], 1–3 years PD = 35.82 [±6.17], > 3 years PD = 51.65 [±8.43]) (**b**) Mean serum IL-10 concentration in pg/ml (< 1-year PD = 27.97[±5.76], 1–3 years PD = 21.69[±4.29], > 3 years PD = 23.86[±4.21]) (**c**) Mean serum TNFα concentration in pg/ml (< 1-year PD = 12.43[±6.74], 1–3 years PD = 2.5[±4.04], > 3 years PD = 0.98[±2.53]) (**d**) Mean serum NOx concentration in mmol/dm^3^ (< 1-year PD = 10.64[±3.59], 1–3 years PD = 18.47[±2.16], > 3 years PD = 15.26[±5.27]). The results are expressed as mean ± SD. ****P < 0.0001 and ***P < 0.001 were considered significant compared to healthy controls (Mann-Whitney U Test)

An increase in serum IFNγ and IL-10 with varying disease durations (< 1-year PD (*n* = 12); 1–3 years PD (*n* = 30); 3–12 years PD (n = 30)) was overall observed in the PD patients in comparison to healthy controls (*P* < 0.0001) (Fig. 1); Conversely, serum TNFα showed a significant increase at early PD (first twelve months) which gradually declined to near zero with disease duration (*P* < 0.001) compared to healthy controls. A significant increase in IFNγ levels with the increase in PD duration (3–12 years of PD) was evident compared to individuals with other neurological disorders (P < 0.001).

A higher level of NOx was noted during 1–3 years of PD with a subsequent decline with increase in durationof PD (*P* < 0.001). However, individuals with other neurological disorders exhibited the lowest levels of serum NOx (Fig. 1).

### Th1:Th2 cytokine ratio

**Fig. 2:**
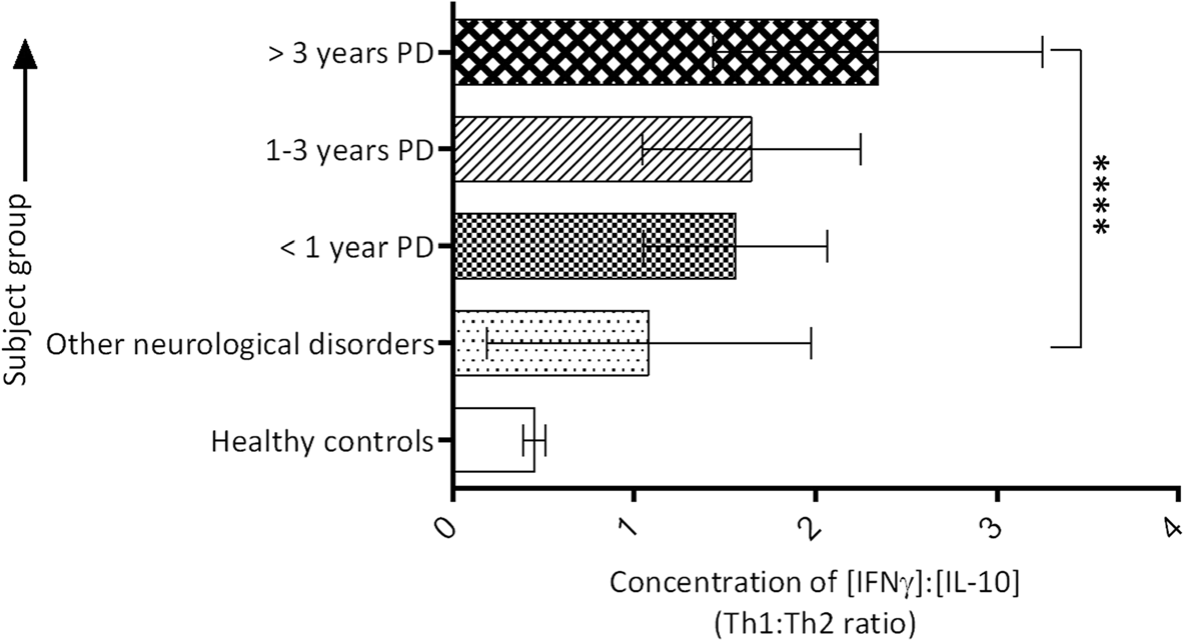
Th1:Th2 serum cytokine ratio (IFNγ:IL-10) in PD patients of varying disease durations, and in controls. Each Th1:Th2 ratio was calculated using individual serum concentrations of IFNγ and IL-10 to obtain the mean ratio of each PD patient for statistical analyses. (Mann-Whitney U Test *P < 0.0001)

The ratio of serum concentrations of Th1: Th2 cytokines, represented by IFNγ:IL-10, increased sequentially with disease duration in PD and was higher than of other neurological disorders and healthy controls. The ratio was the highest in PD patients of 3–12 years compared to PD patients < 1 year of disease history (*P* < 0.0001), but this difference between PD patients of < 1 year and 1–3 years was not significant. The individuals with other neurological disorders manifested a significantly lower ratio than all of the three PD cohorts (P < 0.0001), but was significantly higher than of the healthy controls (*P* < 0.0001) (Fig. 2).

### Association/correlation between PD complications and tested analyte concentrations

Significant correlation evident between serum IFNγ and TNFα levels in both healthy controls (Spearman r = − 0.569; *P* < 0.001) and individuals with other neurological disorders (r = 0.469; *P* < 0.001) was absent in PD patients (< 1 year PD, r = 0.212, *P* > 0.05; 1–3 years PD, r = 0.223, *P* > 0.05; 3–12 years PD, r = 0.068, *P* > 0.05).

Significant associations were established between postural instability and serum TNFα levels (P < 0.001) and between postural dizziness and serum NOx levels (P < 0.001), while low serum NOx levels was associated with cognitive impairment (P < 0.001).

Although, there was an increase in mean H-Y stage with the increase in PD duration (< 1 year PD = 1.36 ± 0.45; 1–3 years PD = 2.24 ± 0.96; 3–12 years PD = 2.38 ± 1.36), this was not statistically significant (*P* > 0.05). In addition to their disease duration of first clinical diagnosis, the serum levels of these immune mediators in all PD patients were analysed based on their H-Y stage. None of the serum immune mediators showed any significant difference with respect to increase in H-Y stage except for TNF which intially showed an increase in patients with mild PD (H-Y scale < 2) and later showed an approximate downward trend although not significant (P > 0.05) as shown in Table 2.

**Table 2:** Serum levels of cytokines and NOx in PD patients based on H-Y stage

H-Y scale	No. of patients	IFNγ	IL-10	TNFα	NOx
No signs of disease (0)	5	55.30 (±15.78)	25.69 (±4.58)	1.52 (±1.74)	16.53 (±6.25)
Unilateral disease (1)	11	40.66 (±6.69)	25.30 (±5.67)	4.97 (±6.13)	12.60 (±3.32)
Unilateral disease plus axial involvement (1.5)	5	46.60 (±8.24)	20.21 (±3.66)	10.30 (±12.29)	15.75 (±7.31)
Bilateral disease (2)	19	42.67 (±8.93)	23.88(±4.76)	3.53 (±4.94)	16.48 (±5.73)
Bilateral disease with recovery on pull test (2.5)	1	43.18 (±0)	22.17 (±0)	1.30 (±0)	25.24 (±0)
Mild to moderate bilateral disease (3)	22	41.76 (±9.99)	22.31 (±4.19)	2.65 (±3.314)	16.60 (±3.36)
Severe disability; still able to walk or stand unassisted (4)	6	54.04(±15.31)	25.14 (±7.37)	1.19 (±2.31)	13.33 (±1.84)
Wheelchair bound or bedridden unless aided (5)	3	40.15 (±7.61)	22.53 (±3.26)	−2.97 (±1.27)	18.58 (±3.38)

All data are presented as mean ± standard deviation. The levels of cytokines are given in pg/ml whereas NOx levels are given in mmol/dm^3^. *P* < 0.05 was considered to be significant; Kruskal-Wallis H test

### CombiROC performance analyses for optimal marker combinations

The tested four analytes as multiple marker signatures, were assessed in different comboson CombiROC and the best combination(s) with the highest area under curve (AUC), sensitivity (SE) and specificity (SP) were selected (Table 3).

**Table 3:** Performance of tested analytes as best single and multiple biomarker panels under ROC curve analysis

Test Group	Marker (s)	AUC	SE %	SP %	Optimum Cut-off
< 1 year PD	IFNγ	0.817	91.7	71.4	0.198
	IL-10	0.731	100.0	58.9	0.179
	TNFα	0.794	91.7	62.5	0.140
	IFNγ-IL-10	0.859	100.0	75.0	0.173
	IFNγ- TNFα	0.862	83.3	78.6	0.266
	IFNγ-IL-10 & TNFα	0.868	83.3	80.4	0.232
1–3 years PD	IFNγ	0.692	93.3	53.6	0.290
	IFNγ-IL-10	0.665	96.7	53.6	0.309
	IFNγ-NOx	0.797	100.0	51.8	0.192
	IFNγ, IL-10 & NOx	0.847	96.7	66.1	0.192
3–12 years PD	IFNγ	0.910	96.7	82.1	0.355
	IFNγ-IL-10	0.920	96.7	89.3	0.429
	IFNγ-NOx	0.924	93.3	87.5	0.416
	IFNγ, IL-10 & NOx	0.924	93.3	87.5	0.408

ROC curve combines sensitivity (SE) and specificity (SP) of a given marker for a diagnostic test from 0.5 (no discriminating power) to 1.0 (complete separation). The combos with high area under curve (AUC), SE and SP were considered as best performing marker ensembles generated from the combinatorial analysis, while those with small SE and SP were considered to be of negligible importance

The diagnostic / prognostic accuracy of individual and of multiple marker combination(s) were calculated by uploading the original data of analyte concentrations to the CombiROC tool. The uploaded data were not subjected to a pre-processing step to preserve their original structure. A test cut off value of 41.2 (control mean + 2SD) was used, since control mean + 3SD did not provide reliable outcomes. The minimum number of positive markers was designated as ‘1’ in the combinatorial analysis step. All possible marker combinations with respect to each PD group were obtained separately by setting the minimum SE and SP values in the next step and finally the best performing individual marker(s) and combos (gold combinations) were obtained via ROC analysis (Table 3).

**Fig. 3:**
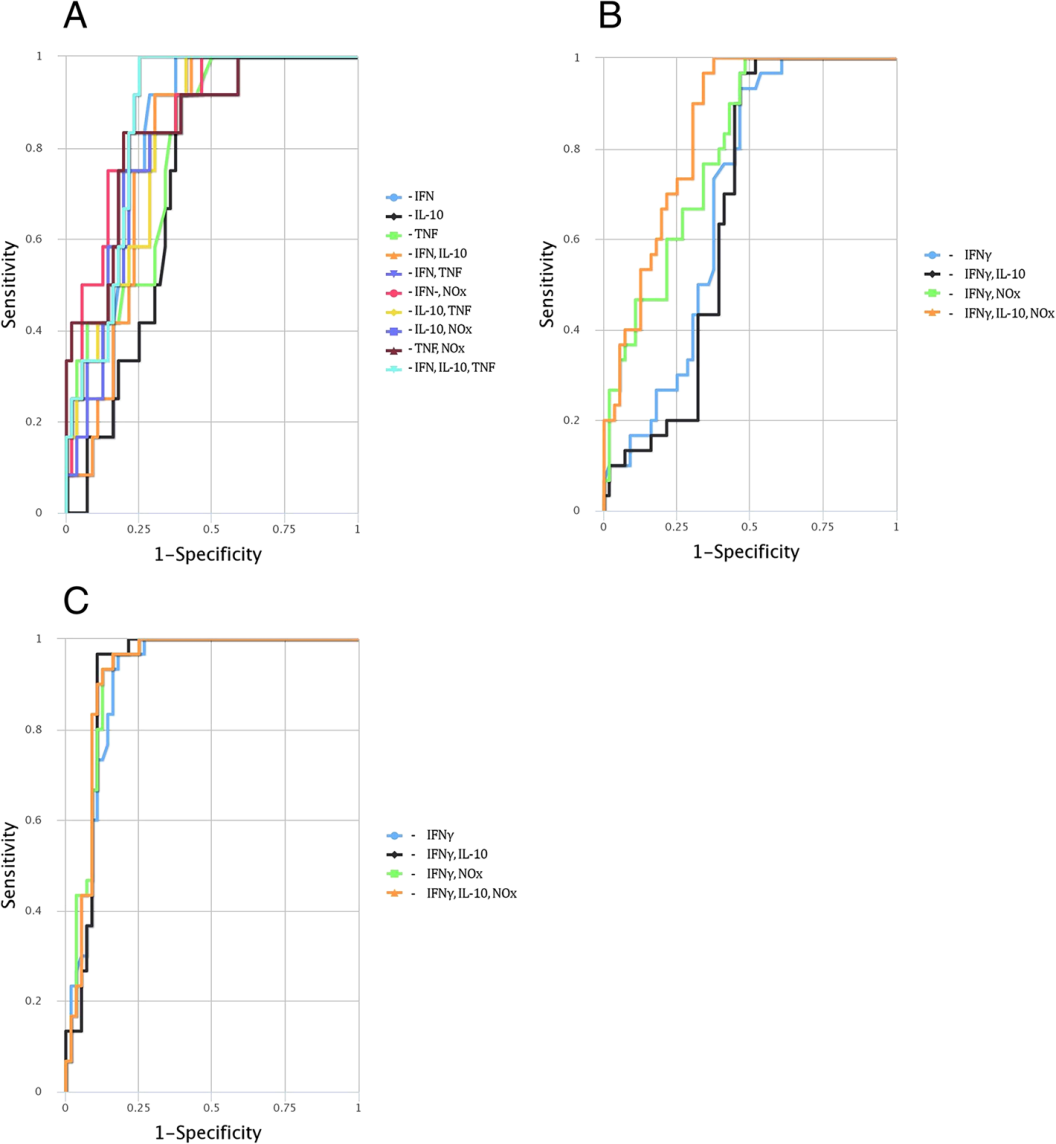
ROC curves for comparing multiple markers and their combinations in PD groups of varying durations.(**a**) less than 1 year PD, (**b**) 1–3 years of PD and (**c**) 3–12 years of PD. ROC curve depicts true positive rate (sensitivity [SE]) on y-axis as function of false positive rate (1 – specificity [SP]) on x-axis. Higher y values correspond to higher SE, whereas lower x values correspond to higher SP

Among the gold combinations, IFNγ performed well above the minimum SE/SP values for all PD groups as an individual biomarker, whereas except for early PD (< 1 year PD), the other individual serum cytokines, IL-10 and TNFα, were not included in the gold combinations. Interestingly, the multimarker panel, IFNγ, IL-10 and TNFα performed well above the minimum SE/SP values (83.3 and 80.4%) compared to controls for early PD (< 1 year of symptom onset) that gradually shifted to IFNγ, IL-10 and NOx (SE = 93.3% and SP = 87.5%) for late PD (1–3 years and 3–12 years of PD) depicting the discriminatory value of TNFα and NOx in early and late PD (Table 3; Fig. 3 (a),(b), (c)).

**Fig. 4:**
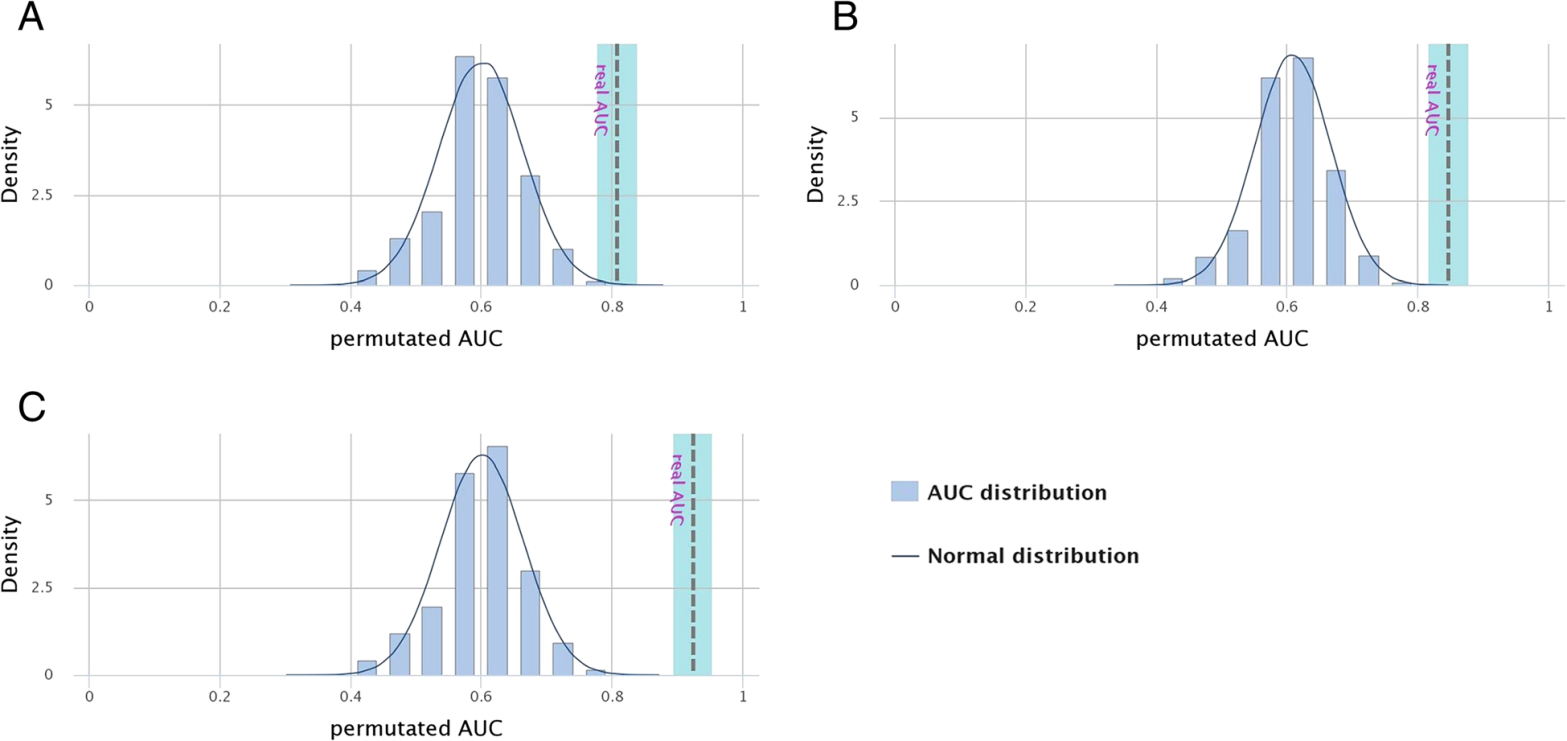
Density distribution of permutated AUC values compared to the normal distribution. The significance of real AUC value for (**a**) less than 1 year of PD, (**b**) 1–3 years of PD and (**c**) 3–12 years of PD. The‘real AUC values’ lie outside the permutated AUC distribution that symbolise high validity of the marker panels generated via CombiROC

A ten-fold cross validation (CV) provided a reliable estimate on the overall panel performance owing to effective clinical validation of the panel; however, since CV yields over-optimistic results, a permutation test was subsequently carried out. A permutation test, in simple terms reassumes the distribution of a particular data set by resampling the observed data. The results obtained (Table 4) suggested that the panel was a perfect fit as the overall accuracy, sensitivity and specificity were least affected by the imposed likelihood (Fig. 4 (a), (b), (c)). In the permutated models, the ‘real’ AUC values were found outside the reference density distribution in all PD groups, rendering them models with high validity (Fig. 4).

**Table 4:** Comparison of performance of the two datasets (10-fold cross validation [CV] and permutated model), generated using CV test for the best combos for the 3 PD groups

Duration of PD	Dataset type	ACC	Error rate	SE (%)	SP (%)	AUC
< 1 year PD	10 fold CV	0.809	0.191	83.3	80.4	0.868
	Permutated model	0.632	6.177	62.5	66.2	0.615
1–3 years PD	10 fold CV	0.744	0.256	96.7	62.5	0.817
	Permutated model	0.626	7.939	62.0	64.7	0.608
3–12 years PD	10 fold CV	0.884	0.116	93.3	85.7	0.904
	Permutated model	0.616	7.844	60.4	65.2	0.605

*ACC* Accuracy, *SE* sensitivity, *SP* specificity, *AUC* area under curve

This compares the performance of our data with a permutated model (where the analysis was repeated N times) with a new data set on the y-axis to estimate new AUC values to provide an estimation on the overall panel performance with higher SE and SP

## Discussion

We report here for the first time that a Th1 cytokine-biased, pro-inflammatory immune response predominates with longer duration of Parkinson disease (PD).

Our data suggest that subtle changes in the peripheral micro-environment of the immune system influence PD, where serum cytokines IFNγ and IL-10 are involved in PD progression whereas TNFα mediated microglial neurotoxicity occurs in early PD. Most recent studies provide proof that neuroinflammatory microglia activates a subtype of astrocytes, A1 via TNFα, IL-1α and complement factor C1q that is extremely neurotoxic to prompt rapid death of dopaminergic neurons in PD [24]. It was postulated that damage associated molecular patterns (DAMPs) released during microglial activation may contribute to the increase in oxidative stress in PD individuals. Thus, it can be hypothesised that oxidative stress occurs secondary to TNFα-mediated microglial neurotoxicity, consequently to the release of DAMPs.

Previous studies on the peripheral cytokine profile in serum of PD patients (including IL-2, IL-4, IL-6, IL-10, TNFα and IL-1β) were suggestive of complex neuropathologic changes occurring in the nigrostriatal pathway and cerebrospinal fluid (CSF) of PD individuals [7–11]. The current study confirms previous reports on the peripheral cytokine profile regarding IL-10, IFNγ and TNFα that demarcated a sudden upsurge in TNF of patients during early PD (less than 1 year of symptom onset/clinical diagnosis). Evidence of neuroinflammatory involvement in PD pathogenesis has been speculated at molecular level but further confirmation is required to comprehend whether these changes are due to activated lymphocytes linked with a continuous inflammatory response, leading to exaggerated cytokine production. The elevated cytokine level may also be indicative of direct efflux of these inflammatory mediators via a disrupted blood-brain barrier (BBB) [8]. Pre-eminent levels of free radicals (NOx) in serum, with longer PD duration (3–12 years PD) symbolise an inflammatory response via inflammatory mediators also confirms that PD is driven by chronic inflammation. This increase in oxidative stress that later decrease with PD duration may be due to the initiation of an active inflammatory response in PD. Clear patterns have emerged from our data on Th1-biased cytokine-mediated immune response in PD, driven by IFNγ and TNFα presumably hastening the neurodegenerative process. Our data suggests the loss of association between IFNγ and TNFα in PD that may be symbolic of loss of their therapeutic effect in activating macrophages. The counter-balancing effect of M1 and M2 macrophages [25] may be lost during PD, leading to a self-propelling chronic inflammation worsening the disease.

Patients with PD manifest both motor and non-motor symptoms related to dopamine and other neurotransmitter deficits [26]. Of the non-motor symptoms, postural instability showed a significant association with serum TNFα levels while postural dizziness showed a significant association with serum NOx levels. A small cross-sectional study performed between PD individuals and normal healthy controls, reported the association between TNFα and non-motor symptoms [27]; Menza et al [28] state that, TNFα is involved in the initiation of important non-motor symptoms in PD, and this was found in our study, denoted by the sudden rise in serum TNFα in early PD (less than 1 year PD) that subsequently ebbed down with duration of PD. However, additional studies are required to confirm whether inflammation-derived oxidative stress contributes to a more aggressive disease course in PD. Both TNFα and NOx being mediators of inflammation, may be considered as a promising novel therapeutic target in treating non-motor symptoms of PD.

Further investigation of these complex interactions is necessary to reveal important clues as to how this neural-cytokine network is involved in the pathophysiology, course and severity of PD. Additionally, outcomes of the current study may provide evidence on peripheral dysregulation in the cytokine network of the immune system in PD, which renders the possibility of manipulating the cytokine network to devise novel approaches to treat PD by targeting to block the cytokine synthesizing pathways that are involved in microglial and astrocyte activation such as NF-κB and JAK-STAT pathways. Similarly, a possibility exists of linking these aggravated immune responses to specific PD states, to develop biomarkers for refining current diagnostic and prognostic criteria and for developing putative therapeutics.

We conducted this study adhering to the Standards for Reporting Diagnostic Accuracy (STARD) 2015 [29]. Diagnostic accuracy of any diagnostic test indicates how well this test discriminates between for e.g. health and disease, two stages of a disease etc. [30]. This discriminative ability can be quantified by the measures of diagnostic accuracy such as the area under curve (AUC), sensitivity, specificity, and predictive values of Receiver Operating Characteristic (ROC) curves. The AUC estimates the discriminative power of the test and indicates in a single value, the overall diagnostic accuracy of the index test. These single biomarkers, once combined as multiple biomarker signatures, can be used to improve the diagnostic accuracy in identifying diseased individuals. We utilized CombiROC [18], a novel concept, in assessing the combined performance of all tested analytes. We focused on identifying cytokine and NOx signatures of different PD durations to further strengthen our claim that immune mediated inflammatory markers play and important role in PD pathogenesis and prognosis rather than on diagnosis.

We generated all possible marker combination(s)/combos for each PD group with respect to controls. The combos that provided the lowest sensitivity (SE) and specificity (SP) values were considered of negligible importance from a clinical perspective. Theoretically, all possible combos (other than those having both SE and SP set at 0) can be used in analyses. However, only the best performing combinations were considered for selection of an ensemble of efficient biomarkers. The multi-biomarker panel, IFNγ, IL-10 and TNFα produced the highest values for SE, SP and AUC for early PD individually as well as combinatorially. Derivatives of nitric oxide (NOx) did not perform well as a single biomarker for late PD; but collectively with IFNγ and IL-10, NOx may be used as a potential multiple biomarker signature for early and late PD. In summary, the combinatorial ROC curve analyses clearly depicted that these immune mediators as a panel, performed as a better indicator/predictor of PD than a single immune marker used in a stand-alone fashion. These data also support our claim that, inflammatory targets should be considered when exploring treatment of non-motor manifestations of PD.

In a previous study that investigated serum immune markers in newly diagnosed PD patients in a large cohort (*n* = 262), showed that the immune marker profile in early PD was associated with cognitive impairment and could be used in predicting future rate of motor impairment. This study further reported that serum levels of cytokines, TNFα, IL-10, IL-2 and IL-1β were higher in newly diagnosed PD patients compared to age and gender-matched controls [31].

However, no previous studies are on record of the accuracy of TNFα, IFNγ and IL-10 and NOx as serum immune biomarkers in PD diagnosis and prognosis, except for their upregulation during PD, these findings require further validation with significanlty larger numbers of subjects of similar cohorts. This study has shown the usefulness of inflammatory biomarkers after the onset of clinical disease and suggested that it would be useful in preclinical diagnosis, which requires longitudinal studies of currently healthy cohorts. However, whether PD progression is related to the loss of peripheral immune balance between pro- and anti-inflammatory responses in the systemic immune system remains debatable.

While significant results were obtained with respect to altered levels of immune mediators in serum of PD patients compared to controls, some of the limitations of our study should be addressed. Since time was a limiting factor, these results are clearly exploratory and preliminary due to small sample size which may explain some of the non-significant differences observed in certain subgroups [eg. < 1-year PD (*n* = 12)]. Additionally, the levels of these immune mediators may be influenced by a variety of factors other than PD pathology, including individual genetic variations, medication, dietary preferences, etc. which were not broadly addressed in our study. Future longitudinal studies utilizing larger sample sizes could help to better characterize variations of these immune markers observed in serum levels and elucidate their relationship underlying PD severity. Furthermore, since no previous studies have been published on clinical performance of immunologic markers in PD diagnosis and prognosis except for their up-regulation during the disease course, additional studies with a large panel of immune markers are warranted for further validation.

## Conclusion

In summary, a Th1 cytokine-based immune response predominates with duration of Parkinson disease. TNFα-mediated neurotoxicity occurs in early PD. This study suggests that serum immune biomarker panel, IFNγ, IL-10 and TNFα, may be proposed as a potential state/diagnostic multi-biomarker panel of PD; in addition, IFNγ, IL-10 and NOx may be suggested as a potential ensemble of rate/prognostic biomarkers of PD progression.

## Additional file

Additional file 1: Interviewer-administered questionnaire for PD individuals. (PDF 117 kb)

## Abbreviations

AUC: Area under the curve
CNS: Central Nervous System
CSF: Cerebrospinal fluid
CV: Cross validation
ELISA: Enzyme-Linked Immuno Sorbent Assay
FBS: Fetal Bovine Serum
H-Y: Hoehn & Yahr
IFNγ: Interferon-γ
IL-10: Interleukin-10
MoCA: Montreal Cognitive Assessment
NOx: Nitric oxide derivatives
PBS: Phosphate Buffered Saline
PD: Parkinson disease
ROC: Receiver Operating Characteristic
RT: Room temperature
SE: Sensitivity
SP: Specificity
Th1/Th2: T helper subset 1/ T helper subset 2
TNF-α: Tumor Necrosis Factor-α
