# Supplementary material for: Selected serum cytokines and nitric oxide as potential multi-marker biosignature panels for Parkinson disease of varying durations: a case-control study
Source: BMC Neurol. 2019 Apr 6;19:56. doi: 10.1186/s12883-019-1286-6 (PMC6451214; doi:10.1186/s12883-019-1286-6)
Supplement: Supplementary file 1 — Interviewer-administered questionnaire for PD individuals. (PDF 117 kb) [file 12883_2019_1286_MOESM1_ESM.pdf]

Immunologic markers in the progression of Parkinson disease

Questionnaire for the survey of demographic data, medical history, disease status and other factors of Parkinson and non-Parkinson individuals in the Colombo District, Sri Lanka.

- This survey is carried out for an academic research by an undergraduate of University of Colombo.
- Confidentiality of all information is carefully guaranteed, and no information by which you can be identified will be released, presented or published. The data will never be used in such a way that you could be identified in any way.

Patient ID no: …………………….

Date: …………………..

1. **Subject Group**

Parkinson Disease (PD) ☐

Other neurological disorders ☐

Normal healthy individuals ☐

Demographic data

2. **Age:** …………years
3. **Sex :** Male / Female
4. **Residential address and Contact details**

…………………………………………………………………………………………………
…………………………………………………………………………………………………
…………………………………………………………………………………………………

5. **Highest educational level**

   GCE Ordinary Level………………..

   GCE Advanced Level………………

Graduate…………………………….
Other (Specify)………………………

6. **Occupation (past / present)**

………………………………………………………………………………………

7. **Past medical history**

<u>Chronic diseases and duration</u>

Diabetes mellitus………………

Hypertension…………………..

IHD…………………………....

Malignancies…………………..

Neurological diseases (specify)…………………………………

Other (specify)…………………………………………………..

Tobacco smoking – pack years (current or past)………………..

Alcohol intake – units per week x years (current or past)..

8. **Drug history**

Name of drug	Start date
………………………….	………………………….
…………………………..	………………………….
………………………….	………………………….
………………………….	…………………………..

9. **Family history of PD**

………………………………………………………………………………………………
………………………………………………………………………………………………

10. Parkinson disease

Date of onset of symptoms: ……………………

Duration since onset of symptoms: ………………………………………

Date of diagnosis: …………………………………

Duration since diagnosis: …………………………………………………..

Diagnosed by Physician / Neurologist / other (specify): ……………………………………..

11. Motor symptoms

**Motor symptoms**	**Right**	**Left**	**Bilateral**
Tremor			
Rigidity			
Bradykinesia			
Postural instability			
Reduced arm swing			
Micrographia			
Mask face			
Freezing of gait			
Dyskinesia			

12. H-Y scale of disease

**Hoehn and Yahr stage**		
Stage	Description	
0	No signs of disease	
1	Unilateral disease	
1.5	Unilateral plus axial involvement	
2	Bilateral disease without impairment of balance	
2.5	Mild bilateral disease with recovery on pull test	
3	Mild to moderate bilateral disease; some postural instability; physically independent	
4	Severe disability; still able to walk or stand unassisted	
5	Wheelchair bound or bedridden unless aided	

13. Non-motor complications

**Non-motor complications**	
Memory impairment	
Hallucinations	
Depression	
Sleep disturbances	
Postural dizziness	
Constipation	
Loss of smell	
